# Survey of maternal sleep practices in late pregnancy in a multi-ethnic sample in South Auckland, New Zealand

**DOI:** 10.1186/s12884-017-1378-5

**Published:** 2017-06-17

**Authors:** Robin S. Cronin, Carol Chelimo, Edwin A. Mitchell, Kara Okesene-Gafa, John M. D. Thompson, Rennae S. Taylor, B. Lynne Hutchison, Lesley M. E. McCowan

**Affiliations:** 1 Department of Obstetrics and Gynaecology, University of Auckland, Private Bag 92019, Auckland, 1142 New Zealand; 2 Department of Paediatrics, University of Auckland, Private Bag 92019, Auckland, 1142 New Zealand

**Keywords:** Stillbirth, Pregnancy, Sleep position

## Abstract

**Background:**

The Auckland Stillbirth study demonstrated a two-fold increased risk of late stillbirth for women who did not go to sleep on their left side. Two further studies have confirmed an increased risk of late stillbirth with supine sleep position. As sleep position is modifiable, we surveyed self-reported late pregnancy sleep position, knowledge about sleep position, and views about changing going-to-sleep position.

**Methods:**

Participants in this 2014 survey were pregnant women (*n* = 377) in their third trimester from South Auckland, New Zealand, a multi-ethnic and predominantly low socio-economic population. An ethnically-representative sample was obtained using random sampling. Multivariable logistic regression was performed to identify factors independently associated with non-left sided going-to-sleep position in late pregnancy.

**Results:**

Respondents were 28 to 42 weeks’ gestation. Reported going-to-sleep position in the last week was left side (30%), right side (22%), supine (3%), either side (39%) and other (6%). Two thirds (68%) reported they had received advice about sleep position. Non-left sleepers were asked if they would be able to change to their left side if it was better for their baby; 87% reported they would have little or no difficulty changing. Women who reported a non-left going-to-sleep position were more likely to be of Maori (aOR 2.64 95% CI 1.23–5.66) or Pacific (aOR 2.91 95% CI 1.46–5.78) ethnicity; had a lower body mass index (BMI) (aOR 0.93 95% CI 0.89–0.96); and were less likely to sleep on the left-hand side of the bed (aOR 3.29 95% CI 2.03–5.32).

**Conclusions:**

Maternal going-to-sleep position in the last week was side-lying in 91% of participants. The majority had received advice to sleep on their side or avoid supine sleep position. Sleeping on the left-hand side of the bed was associated with going-to-sleep on the left side. Most non-left sleepers reported their sleeping position could be modified to the left side suggesting a public health intervention about sleep position is likely to be feasible in other multi-ethnic communities.

**Electronic supplementary material:**

The online version of this article (doi:10.1186/s12884-017-1378-5) contains supplementary material, which is available to authorized users.

## Background

Maternal sleep position in late pregnancy may be a modifiable risk factor for late stillbirth (≥28 weeks) [1]. In the Auckland Stillbirth Study, a matched case control study, women who did not go to sleep on their left side the night before the stillbirth was thought to have occurred, were twice as likely to have a late stillbirth compared with those who went to sleep on their left (adjusted odds ratio (aOR) 2.03 95% confidence interval (CI) 1.24–3.29) [1]. Supine going-to-sleep position was associated with the highest rate of late stillbirth (aOR 2.54 95% CI 1.04–6.18). These findings were independent of other known stillbirth risk factors such as obesity, smoking, and advanced maternal age.

The importance of maternal sleep position and risk of late stillbirth has been confirmed by two subsequent studies [2, 3]. The Sydney Stillbirth Study [3], a matched case control study, reported that women who slept on their backs in the preceding month were six times more likely to have a late stillbirth (aOR 6.26 95% CI 1.2–34). Similarly, a cross-sectional survey of maternal sleep practices and adverse pregnancy outcomes, undertaken in 220 recently delivered women in Ghana [2], reported that supine sleep position during pregnancy was associated with an eight-fold increased odds of stillbirth (OR 8.0 95% CI 1.5–43.2). In addition, interim analysis data from a New Zealand wide case control study of risk factors for late stillbirth reported that the population attributable risk of supine sleep position for late stillbirth was 8.8% [4].

These findings are biologically plausible as supine maternal position in late pregnancy has been associated with decreased maternal cardiac output [5] and uterine perfusion [6, 7], compared with left sided maternal position. In addition sleep disturbed breathing is in general more severe in the supine position [8] and in those with obesity [9], which is also an independent risk factor for late stillbirth [10]. Maternal supine position has also been associated with lower fetal oxygen saturation [11] and changes in fetal cerebral blood flow suggestive of hypoxia [12]. It has therefore been hypothesised that maternal supine sleep position in late pregnancy may be the final stressor leading to stillbirth in vulnerable fetuses with other risk factors [13].

In the Auckland Stillbirth Study, 31% of women in the control group with ongoing pregnancies reported left sided going-to-sleep position on the previous night [1]. More recently, the New Zealand wide case control study has reported that left sided sleep position has almost doubled in the last 3 years to 58%. This suggests that pregnant women are receiving information about optimum sleep position in pregnancy and making changes.

Rates of stillbirth in New Zealand are highest in regions with high deprivation [14]; however, there are no data about maternal knowledge regarding sleep position in these communities. Acquisition of such data will provide important information to assist in developing public health interventions aimed at optimising going-to-sleep position in late pregnancy. Therefore, we undertook a survey of sleep practices and knowledge in a representative multi-ethnic sample of pregnant women residing in Counties Manukau Health (a region predominantly of low socio-economic status) in South Auckland, New Zealand [14]. The aims were to investigate: 1) self-reported maternal sleep position in late pregnancy; 2) knowledge about optimal sleep position; 3) sleep practices; and 4) whether pregnant women believed their going-to-sleep position could be changed to the left side if this was identified as being best for the baby’s health. We also assessed factors related to non-left going-to-sleep position in late pregnancy. We hypothesised that women who slept on the left-hand side of the bed or had received information about sleep position would be more likely to report a left side going-to-sleep position.

## Methods

### Recruitment and data collection

Participants were pregnant women with a singleton pregnancy at ≥28 weeks’ gestation attending community-based antenatal clinics in South Auckland. Eligible women were recruited by research assistants who obtained verbal consent. The self-administered, 30 question survey was undertaken using an online survey software tool, SurveyMonkey® on an electronic tablet and took 10–15 min to complete. The questionnaire is available as supplementary material (Additional file 1). Ethical approval was provided by the University of Auckland Human Participants Ethics Committee (reference number 011116).

### Measures

Maternal demographic data collected included: maternal age, self-identified prioritised ethnicity, country of birth, height and weight, parity, cohabitation with a partner, and the number of people residing in the home. Gestational age on the day of the survey was calculated from the woman’s due date recorded in her maternity record. Prioritised ethnicity was reported according to Ministry of Health Protocols [15].

Weight and height were measured by a research assistant or midwife at the time of interview (87.5%, *n* = 330) and if this information was not available self-reported weight and height were used (12.5%, *n* = 47). Maternal body mass index (BMI) was calculated (weight (kg)/height (m)^2^) and classified according to conventional World Health Organisation [16] criteria. Only two women were classified as underweight with BMI <18.5 kg/m^2^.

Participants were asked what position they usually went to sleep in (over the last week), the reason for their choice of going-to-sleep position, and whether they had received any advice or information about sleep position in late pregnancy. Further questions enquired about the sleep environment: bed size, whether others shared the bed, which side of the bed women usually went to sleep on and why. Women were also asked, “If it was shown that going-to-sleep on your left side in late pregnancy is better for the health of your baby would it be possible for you to change?”

### Power calculation and statistical analysis

In order to recruit a representative sample of women in late pregnancy from the Counties Manukau region with at least 50 women from each main ethnic group (Samoan, Tongan, Maori, Asian, New Zealand European, and Other Ethnicity), a sample size of at least 300 was estimated to be required. Descriptive statistics were used to summarise data such as, demographic characteristics, and sleep position and sleep knowledge in late pregnancy. Multivariate logistic regression was performed to identify factors independently associated with non-left sided going-to-sleep position in late pregnancy. Statistical significance was defined at the 5% level. Analysis was carried out using SAS version 9.3 (SAS Institute Inc., Cary, NC, USA).

## Results

In total, 398 women participated in the study. Of these, 21 women were excluded from the analysis; 18 respondents were less than 28 weeks of gestation and a further three women did not complete most of the survey. Of the 377 completed surveys, height was not recorded for two women, leaving 375 surveys available for multivariable analysis.

Participants had a median age of 27 (SD = 5.5) years and median gestation of 35.7 (SD = 3.5) weeks. The main ethnic groups were Maori, 23.1% (*n* = 87); New Zealand European, 19.6% (*n* = 74); Samoan, 18.3% (*n* = 69); Tongan, 14.1% (*n* = 53); Asian, 14.1% (*n* = 53); Other Ethnicity 10.9% (*n* = 41). Most were born in New Zealand (71.1%, *n* = 268) and lived with a partner (81.4%, *n* = 307). Approximately one quarter (*n* = 100) had no previous children, while 29.4% (*n* = 111) had seven or more people residing in the home. Two-thirds (*n* = 249) of women had a BMI ≥ to 30 at recruitment.

Many participants (61.3%) slept in a queen-size bed and 88.1% reported that they had shared the bed with another person in the last week (Table 1). Similar proportions of women slept on the left-hand (42.4%) and right-hand side of the bed (38.5%). This choice of side of bed was primarily to facilitate getting in and out of bed (39.0%), comfort (35.5%), and habit (32.9%). Almost all (98.1%) women used one or more pillows under their head. The majority (60.5%) reported additional pillows to support their body: 31.6% under their abdomen, 29.2% behind their back, and 25.5% placed a pillow between their knees.

**Table 1:** Self-reported maternal sleep in last week

	Response	*n* = 377 (%)
Size of bed	Queen	231 (61.3)
	King	70 (18.6)
	Double	63 (16.7)
	Single	12 (3.2)
	Floor	1 (0.3)
Shared bed with others	Yes	332 (88.1)
	No	45 (11.9)
Side of bed slept on	Left	160 (42.4)
	Right	145 (38.5)
	Middle	36 (9.5)
	Unsure	36 (9.5)
Reason for side of bed slept on^a^	Easier in or out of bed	147 (39.0)
	More comfortable	134 (35.5)
	Habit	124 (32.9)
	Close to door	54 (14.3)
	Close to bathroom	40 (10.6)
	Children in bed	24 (6.4)
	Partner choice	19 (5.0)
	Close to phone	20 (5.3)
	Other	17 (4.5)
Pillows for sleep^a^	Under head	370 (98.1)
	Supporting abdomen	119 (31.6)
	Behind back	110 (29.2)
	Between knees	96 (25.5)
	Body pillow	6 (1.7)
Usual going-to-sleep position	Either side	146 (38.7)
	Right side	82 (21.8)
	Left side	114 (30.2)
	Sitting/propped	22 (5.8)
	Supine	12 (3.2)
	Prone	1 (0.3)
Reason for usual going-to-sleep position^a^	More comfortable	259 (68.7)
	Easier to get to sleep	120 (31.8)
	Easier to get in or out of bed	89 (23.6)
	Habit	55 (14.6)
	Relieves hip/back pain	29 (7.7)
	Relieves heartburn	24 (6.4)
	Children in bed	20 (5.3)
	Partner choice	14 (3.7)
	Recommended position	15 (4.0)

^a^Percentages add up to more than 100% as some selected more than one reason

The majority (90.7%) reported that they usually went to sleep on their side (left, right or either side) in the last week. The going-to-sleep position was chosen predominantly for comfort (68.7%). Bed sharing with a partner or children was found to have little (3.7 and 5.3% respectively) influence on the reason for sleep position.

Advice from a doctor or midwife about sleep position in late pregnancy was reported by 64.5%. Over half (53.8%) of the 377 women had been advised to sleep on their side or to avoid sleeping supine. Only 50 (13.3%) women recalled being specifically told to sleep on their left side.

Other sources of pregnancy sleep position advice were reported by half the women (50.1%) and this advice was similar to that given by a doctor or midwife. Family and friends were the major source (27.3%), followed by the internet (12.5%), books and pamphlets (8.0%), and childbirth educators (5.8%). Advice accessed from mobile phone applications, radio, TV and newspapers was uncommon (2.4%).

Of the 258 women who had received advice, 12.7% (*n* = 48) changed their going-to-sleep position based on this, including 10 women who were supine sleepers. Thirty five of the 48 women (72.9%) reported they changed to their left side; 54.3% changing from their right side to their left. Five women moved to a propped position and eight to their right side. Most (91.6%) of the 48 women who modified their going-to-sleep position based on advice, reported little or no difficulty making this change.

We asked the 263 non-left side sleepers if it would be possible to modify their going-to-sleep position to their left side if this was of benefit to the baby; most (86.3%) responded that they could change with minimal difficulty (Table 2). Only 5.3% reported that the change would be quite or very difficult. Pillows behind the back (35.3%), between the knees (17.8%), and under the abdomen (11.9%) were considered as potentially helpful aids for over half (59.4%) of the 227 women who reported that change was possible. However, 36 women (13.7% of the non-left side sleepers) reported it would not be possible for them to go to sleep on their left side, even if this was better for the baby; the majority (55.5%) of these women were sleeping on their right side and two (5.6%) reported a supine position.

**Table 2:** Possibility of left side going-to-sleep position if better for baby

Change to left side going-to-sleep position	*n* = 377 (%)
Currently sleep on left side	114 (30.2)
Yes possible	227 (60.2)
No not possible	36 (9.5)
Current going-to-sleep position if change not possible	*n* = 36 (%)
Right side	20 (55.5)
Either side	11 (30.5)
Supine	2 (5.6)
Sitting or propped	2 (5.6)
Prone	1 (2.8)
Anticipated difficulty with changing position	*n* = 227 (%)
Minimal difficulty	213 (93.8)
Quite/very difficult	14 (6.2)
Factors to assist left side going-to-sleep position^a^	*n* = 227 (%)
Pillow or cushion behind back	133 (35.3)
No assistance anticipated	81 (21.5)
Pillow between knees	67 (17.8)
Pillow under abdomen	45 (11.9)
Reminded by partner	43 (11.4)
Change side of bed	33 (8.8)
Change position of bed	23 (6.1)
Change position of partner	14 (3.7)
Children sleeping elsewhere	7 (1.9)
Unsure due to hip pain	2 (0.5)
Change the side of bed slept on to assist left side going-to-sleep position	*n* = 377 (%)
Currently sleep on left-hand side of bed	160 (42.4)
Yes possible	182 (48.3)
No not possible	35 (9.3)

^a^Percentages add up to more than 100% as some selected more than one reason

We also asked about the possibility of changing the side of the bed that women slept on if it would help them change to a left side going-to-sleep position. Of the 217 women who did not usually sleep on the left-hand side of the bed, 83.9% reported that changing to the left-hand side of the bed would be possible.

In the multivariable analysis, four factors were significantly associated with reporting a non-left side going-to-sleep position in late pregnancy (Table 3). These were: Maori (aOR 2.64 95% CI 1.23–5.66) and Pacific (aOR 2.91 95% CI 1.46–5.78) ethnicity compared to Asian and predominantly New Zealand European women; lower BMI (aOR 0.93 95% CI 0.89–0.96); and not sleeping on the left-hand side of the bed (aOR 3.29 95% CI 2.03–5.32). Maternal age, gestational age, living with a partner, and receiving advice on pregnancy sleep position from any source were unrelated to non-left side going-to-sleep position.

**Table 3:** Factors associated with non-left side sleep position (*n* = 375)

Variable	Left side sleep	Non-left sleep	Univariable OR	Adjusted OR
	(*n* = 114)	(*n* = 261)	(95% CI)	(95% CI)
	n (row %)	n (row %)		
Maternal age (years) [mean (SD)]	28.6 (5.7)	27.0 (5.4)	0.95 (0.91–0.99)*	0.97 (0.92–1.01)
Ethnicity (prioritised)				
Maori	23 (26.4)	64 (73.6)	2.12 (1.11–4.05)*	2.64 (1.23–5.66)*
Pacific	43 (27.9)	111 (72.1)	1.96 (1.12–3.45)*	2.91 (1.46–5.78)*
Asian	13 (24.5)	40 (75.5)	2.34 (1.09–5.03)*	1.79 (0.79–4.05)
European & Other	35 (43.2)	46 (56.8)	Reference	Reference
Live with partner				
No	19 (27.1)	51 (72.9)	Reference	Reference
Yes	95 (31.2)	210 (68.9)	0.82 (0.46–1.47)	1.36 (0.70–2.65)
Gestational age (weeks) [mean (SD)]	35.3 (3.6)	35.3 (3.4)	1.01 (0.94–1.07)	1.03 (0.96–1.10)
BMI [mean (SD)]	34.9 (7.6)	32.5 (7.1)	0.96 (0.93–0.99)*	0.93 (0.89–0.96)*
Sleeping on left side of bed				
No	43 (20.0)	172 (80.0)	3.19 (2.02–5.04)*	3.29 (2.03–5.32)*
Yes	71 (44.4)	89 (55.6)	Reference	Reference
Received any advice on sleep position				
No	36 (30.3)	83 (69.8)	1.01 (0.63–1.62)	0.99 (0.59–1.66)
Yes	78 (30.5)	178 (69.5)	Reference	Reference

*OR* Odds ratio, *CI* Confidence interval, *BMI* Body mass index

*Statistically significant (*p* ≤ 0.05)

Multivariable model is adjusted for all the variables in the table

Maternal age, gestational age, and BMI were treated as continuous variables and ORs are for a 1 unit increase

## Discussion

Our survey conducted in a multi-ethnic sample of women in late pregnancy from South Auckland, New Zealand, provides new information about maternal self-reported sleep position, knowledge about optimal sleep position, and whether women could change to a left side going-to-sleep position if there were health benefits for the baby. The large majority (91%) reported that their usual going-to-sleep position in the last week was side-lying. Despite no formal public health education on sleep position in pregnancy, the majority (62%) of participants had received some advice to sleep on their side and to avoid a supine position.

Previous smaller studies [17, 18] have reported that women spend more time sleeping on their left side in late pregnancy than non-pregnant women and that left sided sleeping increases as the pregnancy progresses [18, 19]. This may be due to the abdominal distention of advancing pregnancy causing previous sleep positions to become uncomfortable. Indeed, the majority of women in our survey (69%) chose their going-to-sleep position in the third trimester because of comfort.

In our survey, women reported receiving advice from a doctor or midwife (65%) or media sources (50%) about avoiding supine sleep position or to sleep on their side. Many respondents were therefore aware of the importance of sleep position in late pregnancy. The awareness may be a result of media reports regarding the association of stillbirth and supine sleep following publication of the Auckland Stillbirth Study [1] in conjunction with decades of information about potential consequences of aortocaval compression in the supine position in late pregnancy [5, 6]; especially where there is concern for fetal wellbeing [20, 21], during epidural induction [22], and caesarean section [23]. Furthermore, despite the reported informality and inconsistency of sleep position advice in late pregnancy, 10 participants had changed from the supine going-to-sleep position to a non-supine position based on advice; the majority moving to left side sleeping with relative ease. Although the absolute risk of stillbirths with supine sleep position is relatively low [1], it is possible that a decrease in supine sleep and increase in left sided sleep position in late pregnancy may be a contributing factor to the significant reduction in unexplained stillbirth at term recently reported in New Zealand [14].

We asked women if they could change their going-to-sleep position to their left side if there were health benefits for the baby. Of the 70% who did not currently settle to sleep on their left side, 86% said they could change to left. The large majority (94%) of these women reported that changing would not be difficult for them.

Consistent with findings from another study [18], over half (60%) of participants in our survey reported that they used pillows to support their back, knees and abdomen in their usual going-to-sleep position. Pillows were also the favoured (60%) response when women were asked what might assist those who usually went to sleep in the non-left position to change to their left side, if that was recommended.

Women were asked to identify what side of the bed they usually went to sleep on in the last week. The left-hand side and right-hand side were similar (42 and 39% respectively), followed by middle (10%) and unsure (10%). These figures differed somewhat from an Australian study [18] of 30 women where a greater (57%) proportion reported sleeping on the right-hand side of the bed, less (37%) on the left-hand side, and 7% slept in a single bed.

We undertook multivariable analysis to identify risk factors associated with non-left side going-to-sleep position. The non-modifiable risk factor of ethnicity was identified, which may enable targeting of a future public health intervention on sleep position. BMI was also identified as a risk factor, with heavier women being more likely to sleep on the left side, a finding we are not currently able to explain. A novel and modifiable factor that could become incorporated into a late pregnancy sleep routine, was that sleeping on the left-hand side of bed is associated with a left side going-to-sleep position. Encouragingly, the large majority of participants (84%) who did not currently sleep on the left-hand side of the bed reported that they could change the side of bed they slept on, to help to modify their usual going-to-sleep position from non-left to left, if this was better for the baby.

Maternal age has been previously reported to affect sleep patterns in late pregnancy [18], although in this study, it was not significantly related to choice of left or non-left side going-to-sleep position in the last week. There was also no association between receiving advice on pregnancy sleep position from any source and sleep position, possibly due to inconsistency of the advice received. Furthermore, lack of association with gestation may have been because women in our study were in the third trimester with a median gestation of 35.7 weeks.

### Strengths and limitations

This is the first survey in New Zealand to investigate maternal sleep knowledge, sleep position and influencing factors in late pregnancy. Responses were obtained from a representative sample [14] of multi-ethnic pregnant women in a community predominantly of low socio-economic status. Although maternal sleep position was not able to be validated, participants were provided with a diagram of a bed to aid recall of their sleep position. A moderate correlation between women’s recalled sleep time in the left side position and video evidence has recently been reported [18]. Information bias was reduced by participants and research assistants being unaware of the study objectives. In addition, there has been no formal public health recommendation on late pregnancy maternal sleep position in New Zealand.

## Conclusions

Non-left, and in particular supine, sleep position in late pregnancy may be associated with an increased risk of stillbirth [1–3]. Promoting optimal going-to-sleep position in late pregnancy may therefore have the potential to reduce the incidence of late stillbirth. It was encouraging that most participants reported that their going-to-sleep position in the last week was side-lying. It was also positive that a large majority of women who usually went to sleep in a non-left position in late pregnancy reported that they would be able to change to a left side going-to-sleep position if that was recommended.

The finding that sleeping on the left-hand side of the bed is significantly associated with a left side going-to-sleep position may assist women to more easily modify their sleep position. Furthermore, most women reported that they could change the side of the bed they slept on to achieve this. Our findings have the potential to assist with the development of a public health intervention on how to achieve optimal third trimester going-to-sleep position that can be applied in a multicultural setting.

## Additional file

Additional file 1: Questionnaire. (DOCX 73 kb)

## Abbreviations

aOR: Adjusted odds ratio
BMI: Body mass index
CI: Confidence interval
