# Supplementary material for: Survey of maternal sleep practices in late pregnancy in a multi-ethnic sample in South Auckland, New Zealand
Source: BMC Pregnancy Childbirth. 2017 Jun 17;17:190. doi: 10.1186/s12884-017-1378-5 (PMC5474014; doi:10.1186/s12884-017-1378-5)
Supplement: Additional file 1: — Questionnaire. (DOCX 73 kb) [file 12884_2017_1378_MOESM1_ESM.docx]

**SOUTH AUCKLAND MATERNAL SLEEP IN PREGNANCY SURVEY 2014**

**Uploaded to an online survey software tool, SurveyMonkey®**

**For self-administration on an electronic tablet**

**Study ID: _______________**

**SECTION A: General questions about you and your pregnancy.**

1. **Today’s date**: ______/______/_________ *(day/ month/ year)*
2. **Weight**: ______________ kg  Measured by Interviewer or midwife  Estimated my weight
3. **Height**: ______________ cm  Measured by Interviewer or midwife  Estimated my height
4. **How many babies have you given birth to before this pregnancy**? (Parity): ________
5. **What is your due date**? (EDD): ______/______/________ *(day/ month/ year)*
6. **How old are you**? ___________ *(years)*
7. **Where do you currently live?**

Mangere                        Manurewa               Manukau

Otahuhu                        Otara                       Papatoetoe

Takanini                        Howick                    Botany

Papakura                      Franklin                    Weymouth

Other, please specify___________

1. **Where were you born**? ­

New Zealand  Samoa  China

Australia  Tonga  India

Cook Islands  Niue

Other, specify: _________________

1. **If you were not born in New Zealand, how long have you lived in NZ?** ______ *(years)*
2. **What ethnic group or groups do you belong to?** *(Please tick all options that apply to you)*

New Zealand European

Māori

Samoan

Cook Island Māori

Tongan

Niuean

Chinese

Indian

Other (such as Dutch, Japanese, Tokelauan) *(specify)* _______________________

1. **How many people usually live in your home (including yourself)?** ____________ (*people) Please write the number of adults and children who usually live in your home (including yourself).*

Adults (18 years or older) ________ Children (less than 18 years) ________

1. **Do you live with a partner?**  Yes  No

# SECTION B: Bed

1. **What size bed did you sleep in last night?**  *(Tick* ***one*** *answer only)*

1. King
2. Queen
3. Double
4. King single
5. Standard single
6. Other-didn’t sleep in bed *(specify)___________________________________________*
7. **What size bed did you sleep in last week?**  *(Tick* ***one*** *answer only)*

01  King

02  Queen

03  Double

04  King single

05  Standard single

99  Other-didn’t sleep in bed *(specify)____________________________________________*

1. **Did anyone else sleep in the same bed as you last night?** *(Tick* ***all*** *answers that apply)*

Yes, partner  Yes, child (how many?) __________

Yes, other, specify who _____________  Yes, pet

No-one

1. **Did anyone else sleep in the same bed as you in the last week?** *(Tick* ***all*** *answers that apply)*

Yes, partner  Yes, child (how many?) __________

Yes, other, specify who _____________  Yes, pet

No-one

1. **Which side of the bed did you go to sleep on last night?**

***Looking at the diagram,*** *were you on the:*

*(Tick* ***one*** *answer only)*

1. Left side of the bed
2. Middle of the bed
3. Right side of the bed
4. Unsure


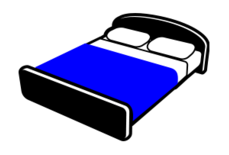


L

R

1. (A) **Which side of the bed did you usually go to sleep on last week?**

***Looking at the diagram****, were you on the:*

*(Tick* ***one*** *answer only)*

01  Left side of the bed

02  Middle of the bed

03  Right side of the bed

99  Unsure

(B**) Why did you choose to sleep on this side of the bed last week?**

*(Tick* ***any*** *answers that apply to you)*

1. Easier to get into or out of bed
2. Closer to bathroom
3. Facing partner
4. Facing away from partner
5. My partner’s preference
6. More comfortable
7. Habit, always gone to sleep this way
8. Close to phone
9. Close to door
10. Close to window
11. Child or children in bed

99  Other, specify

1. **How many pillows did you use**?

| **Pillow placement** | **Number of pillows** | |
| --- | --- | --- |
|  | **Last night** | **Last week** |
| Under your head |  |  |
| Supporting your tummy |  |  |
| Behind your back |  |  |
| Between your knees |  |  |
| Other, specify__________________ |  |  |

**SECTION C: Sleep position**

1. **What position did you usually fall asleep in?**

*(Please circle one answer per line)*

|  | Left side | Back | Right side | Tummy | Position varies | Siting or Propped  Up |  |
| --- | --- | --- | --- | --- | --- | --- | --- |
| Last week | 1 | 2 | 3 | 4 | 5 | 6 |  |
| Last night | 1 | 2 | 3 | 4 | 5 | 6 |  |

1. **Why did you choose this position to go to sleep in?** *(Tick* ***any*** *answers that apply to you)*

Easier to get into or out of bed

Like to face towards my partner

Like to face away from my partner

My partner’s preference

More comfortable

Habit, always gone to sleep this way

Like to face towards or away from door

Like to face towards or away from the window

Relieves reflux /heartburn

Child or children in bed

Easier to get to sleep

Relieves hip or back discomfort

Recommended sleep position

Other, specify ______________________________________________________________

1. **If you lay down on your back in the last two weeks have you felt faint or dizzy?**

Yes  No  N/A (Have not lain on back)

1. **If you stood up quickly from sitting or lying down in the last two weeks have you felt faint or dizzy?**

Yes  No

1. **What position did you usually wake up in to start the day?**

*(Please circle one answer per line)*

|  | Left side | Back | Right side | Tummy | Position varies | Sitting or Propped  up |  |
| --- | --- | --- | --- | --- | --- | --- | --- |
| **Last week** | 1 | 2 | 3 | 4 | 5 | 6 |  |
| **This morning** | 1 | 2 | 3 | 4 | 5 | 6 |  |

1. **(A)**  **What position is the most comfortable for you to go to sleep in?**

Left side

Back

Right side

Front

Propped up

No particular position

**(B) What, if any, advice have you been given by your midwife or doctor about the position you should go to sleep in during late pregnancy (after 28 weeks)?**

*(Tick* ***one*** *answer only)*

01  No information about sleep position

02  Not to be concerned about my sleep position

03  Not to sleep on my back

04  To sleep on either side

05  To sleep on my left side

06  To sleep on my right side

9  Other (specify) _________________________________________________________

1. **(A) Other than information from your midwife or doctor, have you read or heard anything about the best position to go to sleep in during late pregnancy (after 28 weeks)?**

*(Tick* ***one*** *answer only)*

01  No information about sleep position

02  Not to be concerned about my sleep position

03  Not to sleep on my back

04  To sleep on either side

05  To sleep on my left side

06  To sleep on my right side

9  Other (specify) _________________________________________________________

**(B) Where did you get this information?** *(Tick* ***any*** *answers that apply to you)*

01  Radio/TV

02  Newspaper

03  Internet

04  Family and friends

05  Childbirth educator

06  Book or pamphlet

07  Phone app

9  Other (specify) _________________________________________________________

1. **Have you changed the position you go to sleep in in late pregnancy (after 28 weeks) because of the advice or information you have received?**

Yes  No  Have not received advice or information

**If yes, what did you change?**

**Previously went to sleep on:**

Left side  Back  Right side  Front  Propped up  No particular position

**Changed to:**

Left side  Back  Right side  Front  Propped up  No particular position

1. **If you changed sleep position, how hard was it to change?**

*(Tick* ***one*** *answer only)*

Not difficult

A little difficult

Quite difficult

Very difficult

1. **(A) If it is shown that going to sleep on your left side in late pregnancy (after 28 weeks) is better for the health of your baby would it be possible for you to change?**

Yes  No  N/A, already sleep on left side

**(B) If yes, which of the following do you think might help you to go to sleep on your left side?** *(Tick* ***any*** *answers that apply to you)*

Pillow or cushion tucked behind your back to keep you on your left side

Pillow between your knees

Pillow under your tummy

Change side of bed slept on

Ask partner to remind you

Ask partner to change their sleep position

Change position of bed in the room

Have child or children sleep in another bed

No assistance would be required

Other, specify___________________________________________

**(C) If yes, how hard do you think it would be to change?**

*(Tick* ***one*** *answer only)*

Not difficult

A little difficult

Quite difficult

Very difficult

1. **Do you think it would be possible to change the side of the bed you sleep on, if this would help you sleep on your left side?**

Yes  No (If no, why?) ______________________________
